# ClustEHR: a tool for generating synthetic EHR data for unsupervised learning experiments.

**DOI:** 10.23889/ijpds.v7i3.1844

**Published:** 2022-08-25

**Authors:** Nonie Alexander, Daniel Alexander, Spiros Denaxas

**Affiliations:** 1 University Collage London

## Objectives

Clustering algorithms are commonly used to identify disease clusters. New Clustering algorithms are benchmarked on synthetic data to assess their accuracy. These datasets lack the complexities of real electronic health record (EHR) producing a partial assessment of the algorithm. We developed a synthetic EHR cluster generator for benchmarking clustering algorithms.

## Approach

We have created a synthetic EHR cluster generator, clustEHR, based on Synthea, a synthetic EHR generator that produces datasets (with parameterized noise and cluster separation) of known clusters and with clinically relevant patient outcomes. We evaluated clustEHR by generating multiple datasets of variable cluster separation and percentage of noise variables to reflect easy and hard clustering problems. We used a linear model to assess the relationship between these parameters and cluster problem difficulty. K-means accuracy was used as a proxy to measure cluster problem difficulty.

## Results

We have developed a tool for generating synthetic EHR cluster data with clinically relevant outcomes based on the rate of decline of medical observations (e.g. blood pressure). The following parameters are supported: a) number of clusters, b) number of patients in each cluster, c) number and data type of features, d) separation through defining clusters as either diseases such as COPD or dementia (high separability) or inter-disease conditions such as emphysema and chronic bronchitis within COPD (low separability), and e) noise variables through identifying variables not predictive of true cluster outcomes random forest feature importance metric. We show that high cluster separation significantly increases k-means accuracy (coefficient of 0.33). Smaller percent of noise variables increase accuracy though not significantly (coefficient 0.42).

## Conclusion

ClustEHR offers realistic mixed data types as well as outcomes which are frequently used to evaluate clusters when subtyping diseases. The evaluating results suggest that the difficulty of the cluster data can be user determined. The tool can be used to create realistic datasets for evaluating clustering approaches.

